# Dysregulated long noncoding RNAs in the brainstem of the DBA/1 mouse model of SUDEP

**DOI:** 10.1186/s12864-021-07921-7

**Published:** 2021-08-17

**Authors:** Deng Chen, Lina Zhu, Xin Lin, Dong Zhou, Ling Liu

**Affiliations:** Department of Neurology, West China Hospital, Sichuan University, Wai Nan Guo Xue Lane 37 #, 610041 Chengdu, Sichuan China

**Keywords:** Long noncoding RNAs, SUDEP, Epilepsy, DBA/1 mouse

## Abstract

**Background:**

Long noncoding RNAs (lncRNAs) play an important role in many neurological diseases. This study aimed to investigate differentially expressed lncRNAs and messenger RNAs (mRNAs) in the susceptibility gaining process of primed DBA/1 mice, a sudden unexpected death in epilepsy (SUDEP) model, to illustrate the potential role of lncRNAs in SUDEP.

**Methods:**

The Arraystar mouse lncRNA Microarray V3.0 (Arraystar, Rockville, MD) was applied to identify the aberrantly expressed lncRNAs and mRNAs between primed DBA/1 mice and normal controls. The differences were verified by qRT-PCR. We conducted gene ontology (GO), the Kyoto Encyclopedia of Genes and Genomes (KEGG) pathway and coexpression analyses to explore the possible function of the dysregulated RNAs.

**Results:**

A total of 502 lncRNAs (126 upregulated and 376 downregulated lncRNAs) and 263 mRNAs (141 upregulated and 122 downregulated mRNAs) were dysregulated with *P* < 0.05 and a fold change over 1.5, among which Adora3 and Gstt4 were possibly related to SUDEP. GO analysis revealed that chaperone cofactor-dependent protein refolding and misfolded protein binding were among the top ten downregulated terms, which pointed to Hspa1a, Hspa2a and their related lncRNAs. KEGG analysis identified 28 upregulated and 10 downregulated pathways. Coexpression analysis showed fifteen dysregulated long intergenic noncoding RNAs (lincRNAs) and three aberrantly expressed antisense lncRNAs, of which AK012034 and NR_040757 are potentially related to SUDEP by regulating *LMNB2* and *ITPR1*, respectively.

**Conclusions:**

LncRNAs and their coexpression mRNAs are dysregulated in the priming process of DBA/1 in the brainstem. Some of these mRNAs and lncRNAs may be related to SUDEP, including Adora3, Lmnb2, Hspa1a, Hspa1b, Itrp1, Gstt4 and their related lncRNAs. Further study on the mechanism of lncRNAs in SUDEP is needed.

**Supplementary Information:**

The online version contains supplementary material available at 10.1186/s12864-021-07921-7.

## Background

Long noncoding RNAs (lncRNAs) were initially considered a byproduct of RNA polymerase II and as the “noise” of gene transcription without biological function [1]. However, recent studies have described that a large number of lncRNAs have critical roles in chromatin modification and transcriptional and epigenetic regulation [1, 2]. LncRNAs are associated with many physiological processes in the central nervous system, including neurogenesis, regulation of neurotransmitters, ion channels and synaptic plasticity [3]. Specifically, accumulating evidence has demonstrated that lncRNAs are involved in epilepsy. In 2015, Lee et al. confirmed that the expression of lncRNAs is different between epilepsy models induced by kainic acid or pilocarpine and normal controls [4]. More recent findings have also indicated that abnormally methylated lncRNAs and their related downstream pathways are involved in the development and progression of temporal lobe epilepsy [5]. Furthermore, Hsiao reported that targeting the lncRNA SCN1ANAT can improve the seizure phenotype in one animal model of Dravet syndrome [6]. These findings demonstrated that lncRNA studies could provide insight into the mechanism and treatment of epilepsy.

Sudden unexpected death in epilepsy (SUDEP) is one of the most common causes of death in epilepsy [7]. However, its underlying mechanism is largely unknown. The occurrence of SUDEP is highly associated with the brainstem. Patodia et al. reported that in SUDEP postmortem cases, pathological changes have been observed in brainstem respiratory nuclei [8]. Recent MRI studies also identified brainstem volume loss in SUDEP cases [9, 10]. Regarding the molecular mechanism, clinical and laboratory evidence have also pointed out that dysfunction of the serotonin system, adenosine receptors and ion channels in the brainstem are related to SUDEP [11–14]. These findings suggest a brainstem-centered structural basis for SUDEP. More importantly, similar pathological findings have been observed in non-SUDEP death cases with epilepsy [8]. The brainstem of accidental non-SUDEP death in epilepsy cases showed intermediate status between healthy controls and SUDEP, suggesting that repeat seizures could somehow remodel the brainstem [8, 15, 16] and, from a pathological aspect, that structural ‘susceptibility’ to SUDEP may accumulate through this process. Thus, we suspected that epigenetic regulation could participate in this process.

The DBA/1 mouse is a reliable animal model for SUDEP [11]. It is characterized by audiogenic seizures (AGSz), followed by seizure-induced respiratory arrest (S-IRA) and death [17]. Similar sequential events of death immediately following a terminal seizure, which led to respiratory arrest then death, have been observed in SUDEP and near-SUDEP cases [8, 18–22]. Moreover, a variety of studies regarding the DBA/1 mouse SUDEP model have illustrated that several brainstem regions (including periaqueductal gray matter, the respiratory complex and raphe nuclei) are associated with SUDEP [23–25]. Interestingly, the susceptibility to S-IRA of DBA/1 increases when given sound stimulation on PND 21 to 30 (which is called priming). Once primed, such susceptibility will last until PND 100 [26]. This distinct feature of DBA/1 makes it unique compared to other SUDEP models, and epigenetic regulation in this process is also speculated. Thus, studying the priming process on DBA/1 could provide insight into the SUDEP mechanism from an epigenetic perspective.

In light of these findings, we hypothesized that lncRNAs participate in the process of gaining susceptibility of S-IRA in DBA/1 and SUDEP. We investigated the expression profiles of lncRNAs and messenger RNAs (mRNAs) in the brainstem of primed and normal DBA/1 by microarray analysis. Assessing the potential role of lncRNAs in increasing susceptibility to S-IRA may provide new insight into the potential mechanism and therapeutic targets of SUDEP.

## Methods

### Animals

The animal experiments were performed in accordance with the Guidelines of Animal Care and Use Committee of Sichuan University. Male, postnatal day (PND) 21–23 DBA1 mice were obtained from the Charles River Laboratories Experimental Animal Center (Beijing, China) and housed in specific pathogen free (SPF) conditions for 1 week prior to experimentation. The DBA/1 mice had access to food and water and were kept under standard lighting conditions (12-h light/dark cycle).

### Seizure priming and seizure-induced sudden death

DBA/1 mice at 28 PND (10–15 g) were used in the experiments. An electric bell (120 dB SPL) (SCF 8, MinRong’s Electronics, China) was used to induce generalized audiogenic seizures (AGSz) in the experimental group. Each mouse was placed in a 40-cm diameter cylindrical plastic container and was given acoustic stimulus lasting for 60 s or until the onset of a seizure. Some mice could develop seizure-induced respiratory arrest (S-IRA) during seizures. The rodent respirator was used to rescue the mice with S-IRA by resuscitating within 5 s after the final respiratory gasp, as reported previously [26]. We stimulated each mouse three times a day for a week. If any mice presented with S-IRA, the rest of the stimulation on that day was cancelled, and the mice rested for 24 h.

### Grouping

The mice that experienced S-IRAs and were successfully rescued more than three times during priming were considered a SUDEP model. Normal controls were those without acoustic stimulation. At PND 36 (24 h after the last stimulation, all had seizures the last day), the SUDEP model and normal mice (four for each group) were decapitated under anesthesia with isoflurane inhalation, and the brains were removed immediately. We used a sharp surgical blade to separate the whole brainstem between bregma − 3 mm and bregma − 9 mm based on a previous report [23].

### Total RNA extraction

The brainstems were immediately frozen in liquid nitrogen and transferred to a -80 ℃ refrigerator for later use. According to the manufacturer’s instructions, total RNA was extracted using TRIzol reagent (Invitrogen Life Technologies, Carlsbad, CA). A NanoDrop ND-1000 spectrometer (Thermo Fisher Scientific, USA) was used to determine the quantity and quality of extracted RNA. The standard denaturing agarose gel electrophoresis method was applied to measure RNA integrity.

### Microarray analysis

We used Arraystar mouse lncRNA Microarray V3.0 (Arraystar, Rockville, MD), containing 35,923 lncRNAs and 24,881 coding transcripts, to describe the lncRNAs and protein coding transcripts of the mice. Sample labeling and array hybridization were performed following the Agilent One-Color Microarray-Based Gene Expression Analysis protocol (Agilent Technology) with slight changes. After removing rRNA from total RNA (RNA-ONLY Eukaryotic RNA Isolation Kit, Epicenter), purified mRNA was obtained. We amplified each sample into fluorescent cRNA by using random primers (Arraystar Flash RNA Labeling Kit, Arraystar). Then, we used an RNeasy Mini Kit (Qiagen) to purify labeled cRNAs and a NanoDrop ND-1000 to detect the concentration and activity. The labeled cRNA was hybridized onto microarray slides and then washed, fixed and scanned successively.

### Quantitative real-time PCR

Four extra SUDEP models and 4 controls were prepared following the same protocol in "Animals" "Seizure priming and seizure-induced sudden death" "Grouping" for this test. We used TRIzol reagent (Invitrogen Life Technologies) to extract the total RNA from frozen brainstem tissues and SuperScript™ III Reverse Transcriptase (Invitrogen) to transcribe cDNA following the manufacturer’s protocol. Dysregulated lncRNAs were examined by quantitative real-time PCR (qRT-PCR) using the SYBR green PCR kit and the ViiA 7 Real-time PCR system (Applied Biosystems). GAPDH served as an internal control for normalization. A sample of each mouse was tested in triplicate. For each target lncRNA, we first used a tenfold serial dilution (from 1 to 10^6^) to establish a standard curve to optimize the reaction conditions. Amplification primers are shown in Table 1. The differentially expressed lncRNAs in the SUDEP model group were measured by fold change relative to the control group, calculated as follows:

**Table 1:** Primers for qRTPCR

Seqname	Primers (5’3’)	Amplicon size (bp)
uc007urg.1	F: TAGCAGTGGTGCCTGTGAC R: GATGGACTCAGGAGGGTCAT	131
AK029922	F: GTCTTCTCCCGTTGGCTTCTATC R: CTATCTGGGCTTATCTTGAGCAGAT	179
ENSMUST00000152600	F: TAAGCCCTAGATGGATGTGT R: TACCAGTATGGGTCCCTAAA	132
ENSMUST00000172531	F: GCCGTGTTTTCACCCTTCTT R: CACGCCACTGCCGATTTT	281
GAPDH	F: CACTGAGCAAGAGAGGCCCTAT R: GCAGCGAACTTTATTGATGGTATT	144

Note: *F* forward, *R* reverse

$$ \mathrm{Fold}\ \mathrm{change}={2}^{-\left(\varDelta \varDelta \mathrm{Ct}\ \mathrm{SUDEP}-\varDelta \varDelta \mathrm{Ct}\ \mathrm{Control}\right)} $$

### Data analysis

We used an Agilent DNA Microarray scanner (part number G2505C) to scan the hybridized images and Agilent Feature Extraction software (version 11.0.1.1) to analyze the acquired array images. The GeneSpring GX v12.1 software package (Agilent Technologies) was applied to perform quantile normalization and further data processing. After quantile normalization, all intensities underwent log2 transformation for further statistical analysis. The P value was calculated by unpaired t-test based on these normalized and transformed intensities. *P* < 0.05 and fold change ≥ 1.5 were used to identify statistically significant dysregulated lncRNAs and mRNAs. The results were presented by scatter plots. Clustering analysis was performed to show the different expression patterns of lncRNAs and mRNAs in different groups. Gene ontology (GO) analysis was applied to investigate their molecular functions, biological processes and cellular components. Pathway analysis of differentially expressed mRNAs was explored using the Kyoto Encyclopedia of Genes and Genomes (KEGG) database. Coexpression analysis was created to identify differentially expressed antisense lncRNAs with their related sense mRNAs and long intergenic noncoding RNAs (lincRNAs) with their nearby (< 300 kb) coding genes.

## Results

### Overview of differentially expressed lncRNAs and mRNAs

**Fig. 1:**
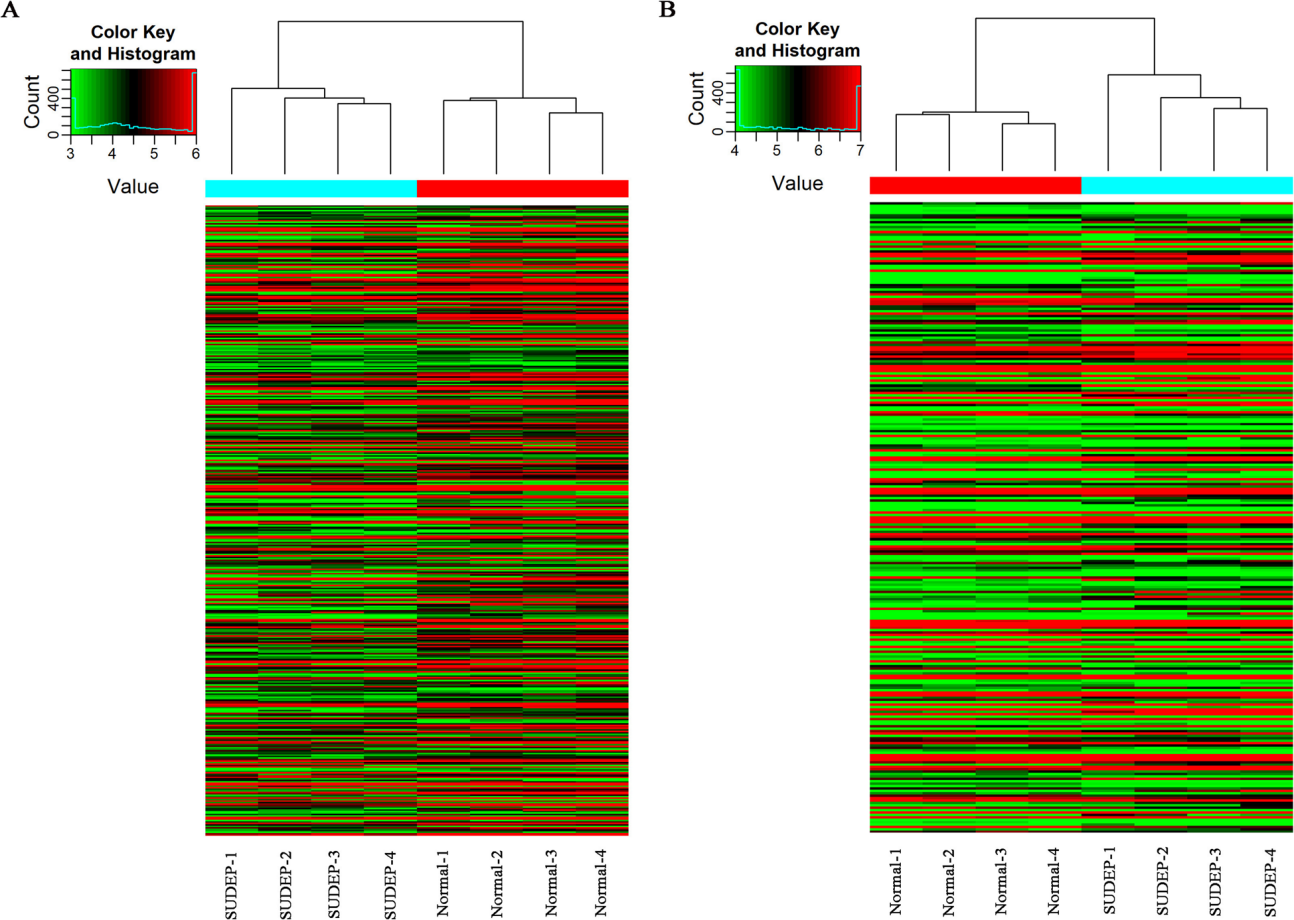
**A** Heat map and hierarchical clustering of lncRNA profile comparison between SUDEP group and normal group; **B** Heat map and hierarchical clustering of mRNA profile comparison between SUDEP group and normal group. Red color indicates high expression and green color indicates low expression

**Fig. 2:**
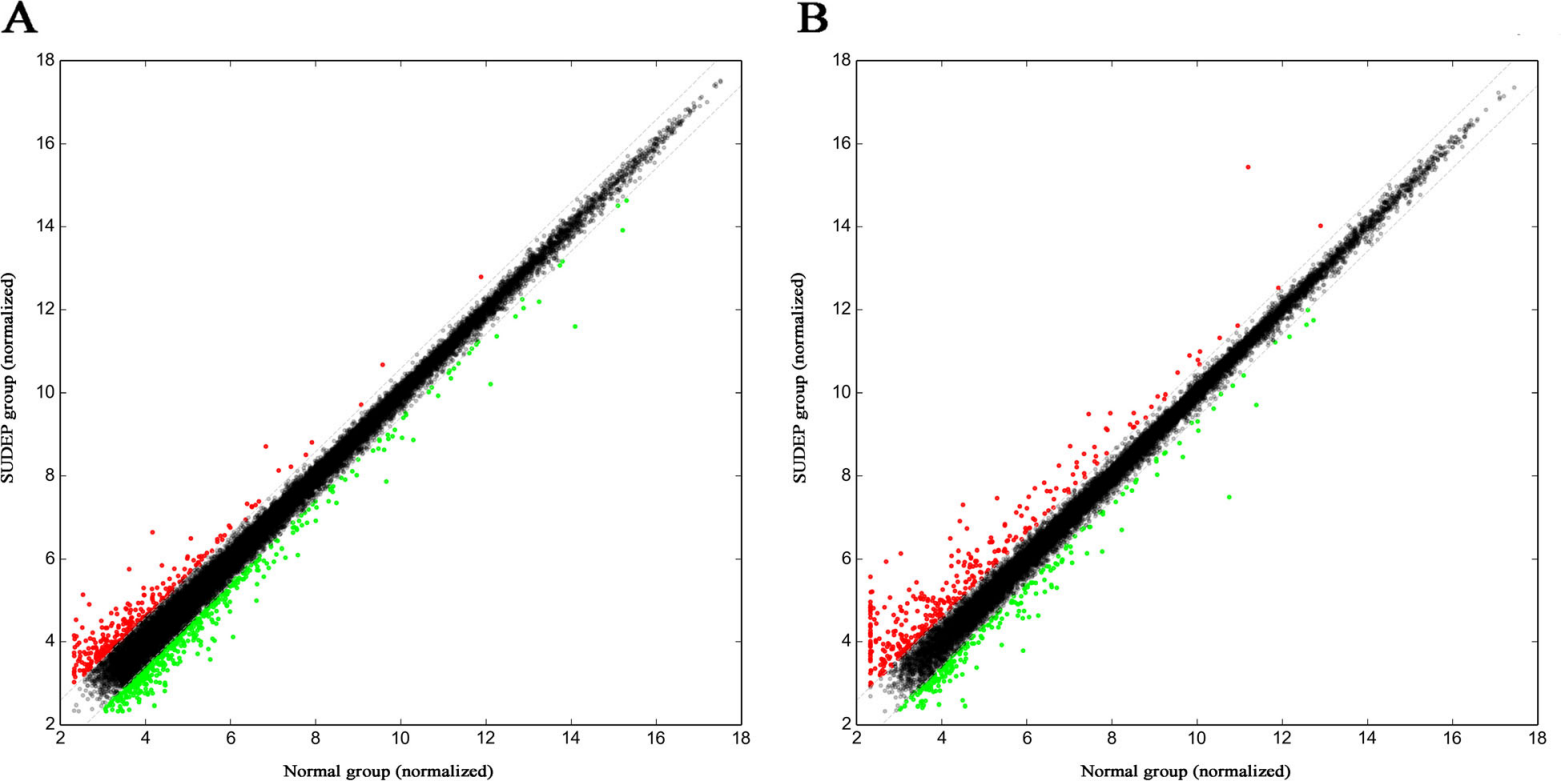
The scatter plots of lncRNA (**A**) and mRNA (**B**) expression between SUDEP group and normal group. The values of the X and Y axes are normalized in the two groups (log2-scaled). The red color and green color above the top grey line or below the bottom grey line represent up- or down-regulated genes respectively, which change more than 1.5 fold (*P* < 0.05) between the two groups

There were 502 lncRNAs and 263 mRNAs found to be dysregulated. A total of 126 lncRNAs were upregulated and 376 lncRNAs were downregulated, while 141 mRNAs were upregulated and 122 were downregulated. A heat map and hierarchical clustering analysis of lncRNAs and mRNAs between normal DBA/1 mice and the SUDEP group are shown in Fig. 1. Scatter plots showing the variation in lncRNA and mRNA expression between the two groups are also depicted in Fig. 2. The top 10 dysregulated lncRNAs and mRNAs are summarized in Tables 2 and 3. Specifically, no mRNA related to serotonin or ion channels (potassium, sodium, and calcium) was found to be different. However, adenosine receptor 3 was downregulated in the SUDEP group (Gene symbol: *ADORA3*, fold change 2.03 down, *P* = 0.043). Other RNAs of special interest included Hspa1a (fold change 9.64 down, *P* < 0.001), Hspa1b (fold change 3.20 down, *P* < 0.001), Itrp1 (fold change 1.54 up, *P* = 0.039), and Lmnb2 (fold change 1.78 down, *P* = 0.002) on the mRNA; NR_040757 (fold change 1.56 down, *P* = 0.037), Gstt4 (fold change 1.51 down, *P* = 0.027), AK012034 (fold change 1.86 down, *P* = 0.025), and ENSMUST00000172531 (fold change 2.60 down, *P* = 0.010) were on lncRNA.

**Table 2:** Top 10 differentially expressed lncRNAs in SUDEP group compared with normal group

Up-regulated lncRNAs				Down-regulated lncRNAs			
Seqname	Gene symbol	Fold Change^a^	Chromosome	Seqname	Gene symbol	Fold Change^a^	Chromosome
uc007urg.1	AK013627	5.5312972	14	uc.352-	uc.352	3.8636426	14
ENSMUST00000118822	Rpl7l1-ps1	4.3902715	X	AK029922	AK029922	3.3637177	6
ENSMUST00000161007	Gm15717	3.6814342	8	humanlincRNA1929-	humanlincRNA1929	3.1524491	10
AK089422	AK089422	3.5432344	15	ENSMUST00000141932	Lrrc57	3.0635987	2
humanlincRNA1945-	humanlincRNA1945	3.3141253	10	AK080458	AK080458	3.0231669	13
AK050947	AK050947	2.8759742	12	ENSMUST00000117916	Gm9850	2.8692668	5
mouselincRNA0735+	mouselincRNA0735	2.8726083	17	ENSMUST00000139399	Rara	2.8357532	11
AK042193	AK042193	2.8206605	1	AK035001	AK035001	2.7316083	10
AK013439	AK013439	2.746032	2	ENSMUST00000172531	1110038B12Rik	2.6065826	17
ENSMUST00000152011	Car12	2.5985299	9	uc007ysi.1	AK020351	2.5099841	16

Notes: ^a^The SUDEP group compared with normal group

**Table 3:** Top 10 differentially expressed mRNAs in SUDEP group compared with normal group

Up-regulated mRNAs				Down-regulated mRNAs			
Seqname	Gene symbol	Fold Change^a^	Chromosome	Seqname	Gene symbol	Fold Change^a^	Chromosome
NM_013697	Ttr	18.8947104	18	NM_010479	Hspa1a	9.6381486	17
NM_007976	F5	9.4551777	1	NM_001159671	Rsph6a	3.7486615	7
NM_001142706	Cfb	7.2709559	17	NM_010478	Hspa1b	3.1997402	17
NM_024283	1500015O10Rik	6.9567191	1	NM_026080	Mrps24	2.7997696	11
NM_010030	Defb2	6.2209284	8	NM_001164259	Fgfrl1	2.7403876	5
NM_020516	Slc16a8	5.5709692	15	NM_177294	Rpap1	2.7259814	2
NM_009902	Cldn3	4.8754256	5	NM_001099328	Zfp831	2.722746	2
NM_139221	Defb11	4.863742	8	NM_026159	Retsat	2.6378435	6
NM_001033233	Tmprss11a	4.7814956	5	NM_013604	Mtx1	2.5202344	3
NM_013823	Kl	4.5972095	5	NM_001163667	Tnnt3	2.4143907	7

Notes: ^a^The SUDEP group compared with normal group

### Gene Ontology (GO) analysis

**Fig. 3:**
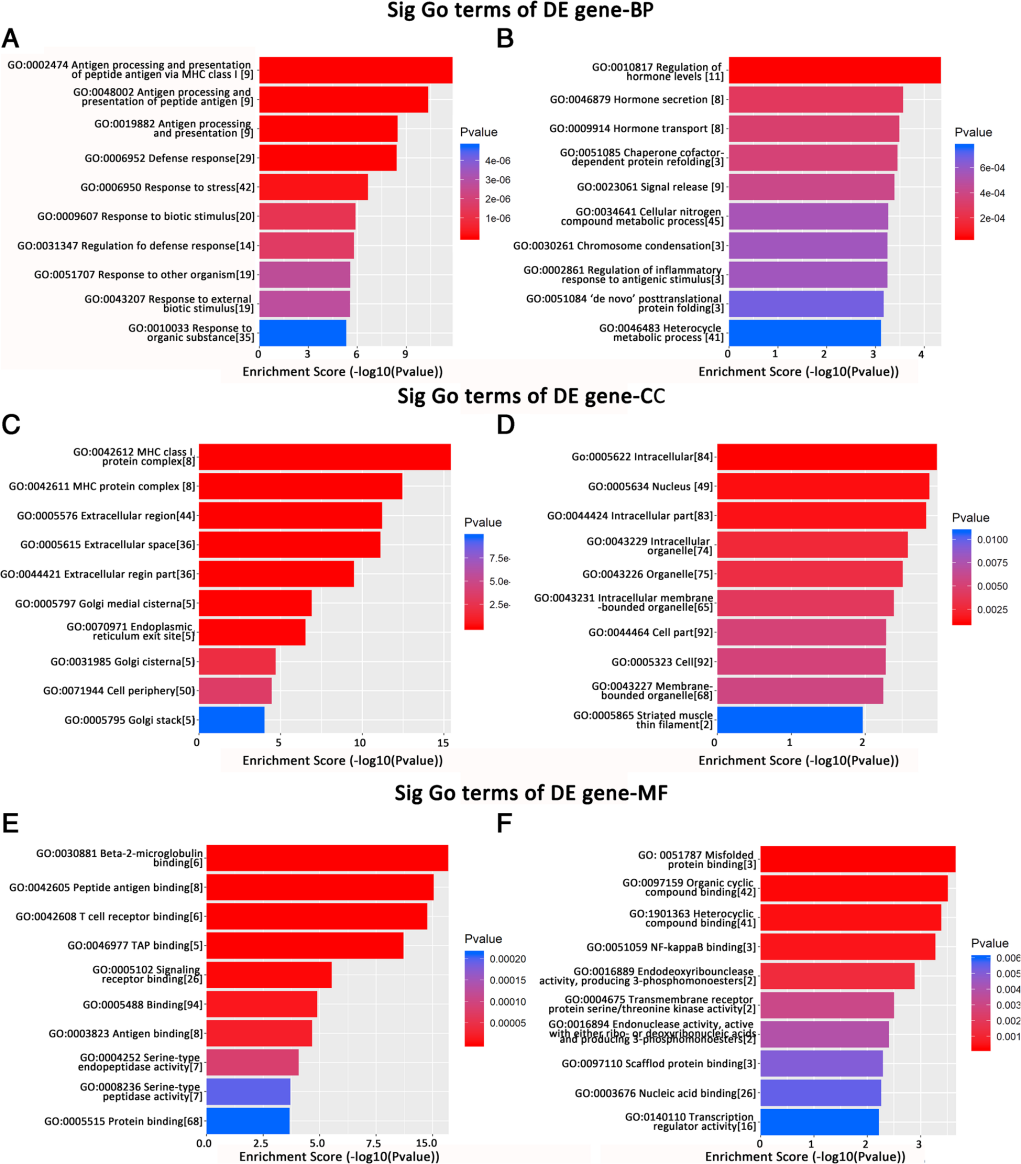
Top 10 enriched GO terms for the up-regulated (**A**) and down-regulated (**B**) mRNAs involved in biological process; Top 10 enriched GO terms for the up-regulated (**C**) and down-regulated (**D**) mRNAs involved in cellular components; Top 10 enriched GO terms for the up-regulated (**E**) and down-regulated (**F**) mRNAs involved in biological process molecular function. The numbers in square brackets stand for the number of genes participating in

To elucidate the function of differentially expressed mRNAs, GO analysis was applied to assess the biological processes, cellular components, and molecular functions. The most enriched GO terms corresponding to the upregulated genes were antigen processing and presentation of peptide antigen via the MHC class (GO:0002474, *P* < 0.001, Fig. 3A) in biological processes, the MHC class I protein complex (GO:0042612, *P* < 0.001, Fig. 3C) in cellular components and beta-2-microglobulin binding (GO:0030881, *P* < 0.001, Fig. 3E) in molecular functions. The top enriched GO terms according to downregulated genes were regulation of hormone levels (GO:0010817, *P* < 0.001, Fig. 3B) in biological processes, intracellular (GO:0005622, *P* = 0.001, Fig. 3D) in cellular components and misfolded protein binding (GO:0051787, *P* < 0.001, Fig. 3F) in molecular functions. This part of the results is presented in Fig. 3.

### Pathway analysis

**Fig. 4:**
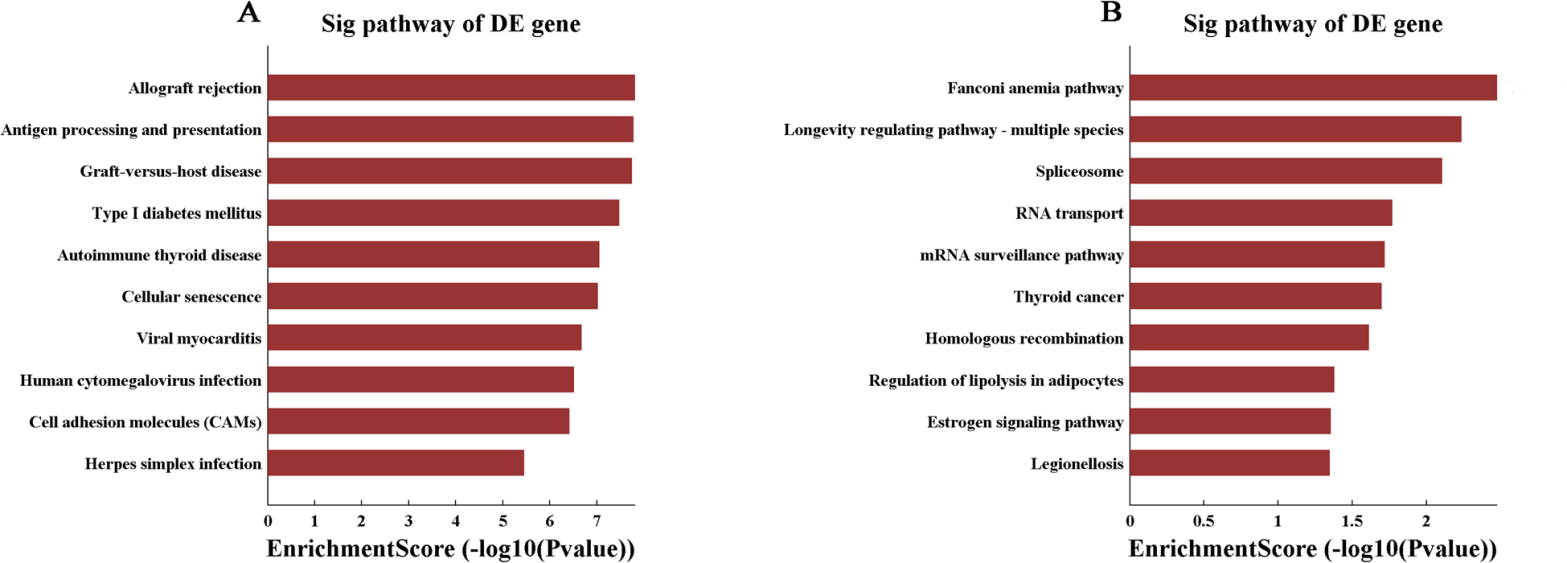
**A** Pathway analysis of upregulated mRNAs in SUDEP group; **B** Pathway analysis of downregulated mRNAs in SUDEP group

Pathway analysis was performed to obtain the biological pathways of the differentially expressed mRNAs based on the Kyoto Encyclopedia of Genes and Genomes (KEGG) database. In contrast with the normal group, we identified 28 upregulated and 10 downregulated pathways in the SUDEP group. For the upregulated genes, the most highly enriched biological process was allograft rejection, while the Fanconi anemia pathway was the top downregulated pathway. The top 10 dysregulated pathways are shown in Fig. 4.

### Coexpression analysis

Three differentially expressed natural antisense lncRNAs (AK012034, AK032393, and uc007ovn.2) and their paired coding genes (Lmnb2, Zmym1, and Ifi27) were identified. The details are described in the supplementary material (see Additional file 1: Table 1).

This study also revealed fifteen dysregulated long intergenic noncoding RNAs (lincRNAs) and their associated mRNAs, of which the most aberrantly expressed lincRNA was upregulated AK013439, and its corresponding mRNAs were Gnas (downregulated) and Zfp831 (downregulated). The results are presented in the supplementary material (see Additional file 1: Table 2).

### Quantitative real-time PCR validation

**Fig. 5:**
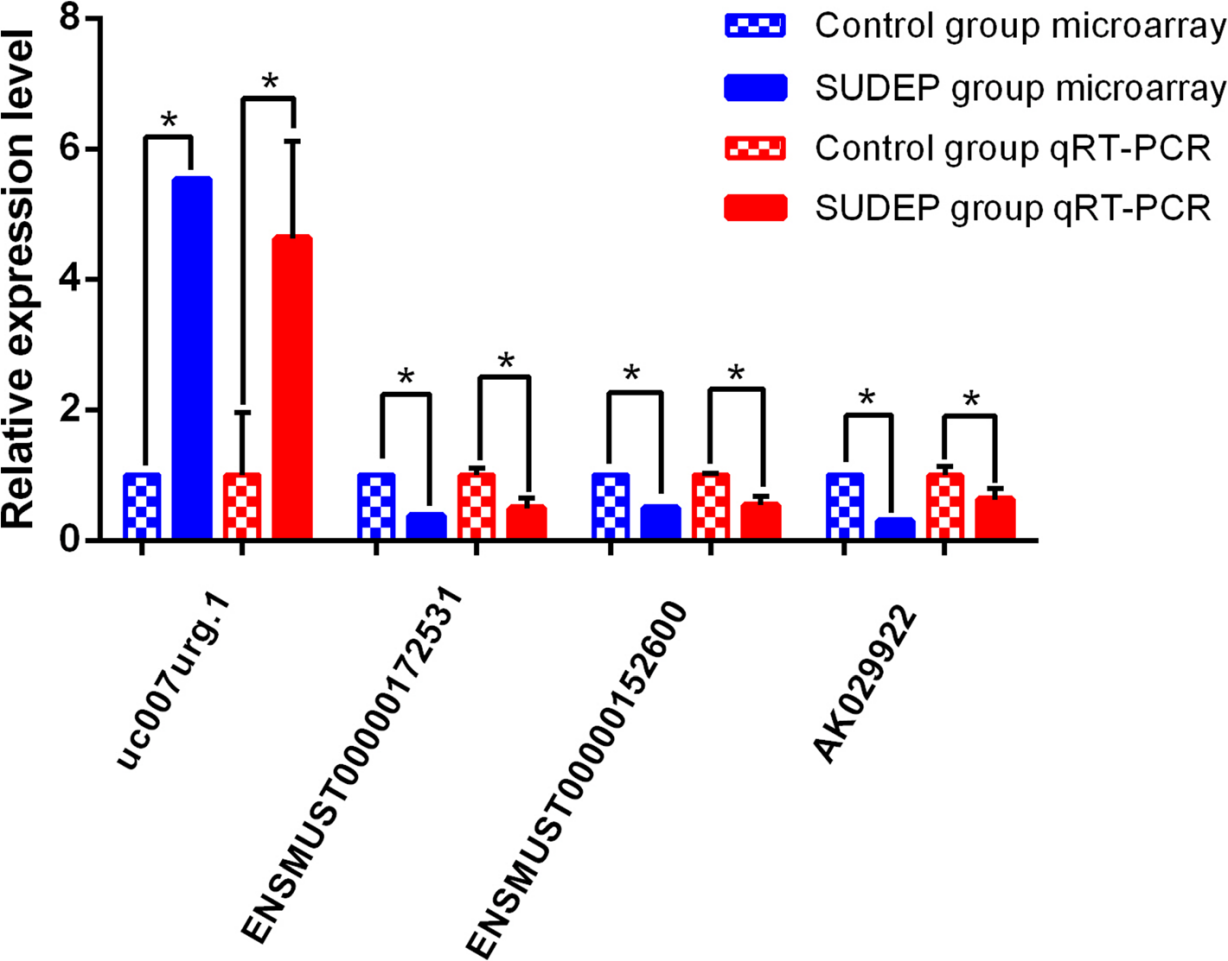
The validation of differentially expressed lncRNAs. Note: The expression value of lncRNAs in control group was set at 1, while the corresponding expression level of which in SUDEP group was the fold change relative to control group. The qRT-PCR results were consistent with microarray data. Significant levels were indicated by * (*P* < 0.05)

Four lncRNAs (uc007urg.1, AK029922, ENSMUST00000152600, ENSMUST00000172531) were selected for further validation by qRT-PCR. The results showed that all these lncRNAs exhibited significant differences (*P* value < 0.05) between the two groups, which was in accordance with the microarray results (Fig. 5).

## Discussion

Over the past decades, an increasing number of studies have explored the potential molecular and electrophysiological mechanisms of SUDEP. Recently, increased awareness has been given to SUDEP-related genes, which have mostly been identified as neurocardiac genes (i.e., KCNA1, SCN1A, SCN8A, KCNQ1) [12, 27]. However, although some SUDEP cases have a genetic background, a large proportion of cases have not carried clear genetic mutations [28]. This suggests that SUDEP is a highly heterogenic disease in genetics and requires further study in different aspects.

However, much less is known about the changes and roles of mRNAs and noncoding RNAs (ncRNAs) in SUDEPs. In Scorza et al.’s study, they first reported that microRNAs might participate in SUDEP [29]. De Matteis et al. identified miR-301a-3p as an innovative potential biomarker in SUDEP [30]. Other ncRNAs, including lncRNAs, are still poorly understood in SUDEP. Inspired by recent findings on pathology that repeat seizures lead to intermediate structural changes between normal brainstem and SUDEP cases [8], we speculated that there is an epigenetic regulation process that alters the structure and function of the brainstem.

To further explore the potential role of lncRNAs and epigenetic regulation in SUDEP, we studied the susceptibility gaining process in a DBA/1 mouse SUDEP model. We found a total of 502 lncRNAs and 263 mRNAs dysregulated between the SUDEP model and normal DBA/1 control. On the mRNA expression profile, we first tried to establish a relation between differentially expressed mRNAs according to known hypotheses related to SUDEP. According to a previous SUDEP mechanism study on this model, fewer serotonin (5-HT) receptors characterized S-IRA-prone mice [23]. However, we did not find any changes in the mRNA of these receptors. This suggests that the difference in the 5-HT system was not made by priming and thus more likely to be intrinsic than epigenetic. Similarly, there was no evidence from our data that any specific ion channel was related to SUDEP. On the other hand, we found significant downregulation of the adenosine A3 receptor. Recently, an imbalance of adenosine receptors in the cortex has been proposed to increase the risk for SUDEP [31]. Adenosine itself is a strong anticonvulsant, while overaccumulation of adenosine could induce respiratory arrest and apnea, thus participating in SUDEP [32]. However, there have been no reports on the relationship of adenosine A3 receptors to epilepsy or SUDEP. In other studies, adenosine A3 receptor has broad functions, including classical functions as a G protein-coupled receptor, inhibiting calcium current and novel functions when binding with N ^6^-methyladenosine (m6A) [33, 34]. Further mechanistic studies are needed to establish its linkage on SUDEP.

In coexpression analysis, both antisense lncRNAs and lincRNAs exhibited differential expression. Antisense lncRNAs serve critical functions in gene expression by regulating transcriptional or posttranscriptional processes [35]. Three natural antisense lncRNAs (AK012034, AK032393, and uc007ovn.2) were found to be differentially expressed in the current study. The antisense mRNA of AK012034 was identified as Lmnb2, which presented a 1.78-fold downregulation in the SUDEP group. It has been reported that a mutation in *LMNB2* could lead to progressive myoclonus epilepsy [36, 37]. This finding implied a potential epigenetic regulation of susceptibility to SUDEP through AK012034 and *Lmnb2*.

LincRNAs are another subgroup of lncRNAs that have been observed to have regulatory roles at the transcriptional and epigenetic levels [38]. However, the function of most lincRNAs remains unknown. Fifteen dysregulated lincRNAs and adjacent coding mRNAs were identified in this study. Among these differences, lncRNA NR_040757 and its nearby gene *ITPR1* could be related to SUDEP, as the mutation of *ITPR1* has been found in SUDEP cases [39]. However, the underlying mechanism requires further study.

GO and KEGG pathway analyses addressed the biological functions of differentially expressed lncRNAs and mRNAs. In GO terms of biological process and molecular function, we focused on chaperone cofactor-dependent protein refolding and misfolded protein binding, which was surprisingly found among the downregulated terms. The related mRNAs are Hspa1a and Hspa1b, and they encode HSP-70 (heat shock protein, 70 kDa). HSP-70 has been tested in a postmortem study of SUDEP cases and has been found to be upregulated in the hippocampus [40]. A recent study of proteomics and RNA sequencing also revealed similar patterns in SUDEP cases and mesial temporal lobe epilepsy (mTLE). HSP-70 was thus considered a marker of recent seizures prior to final death [41]. However, in our study, even though all sacrificed mice suffered from seizures 24 h prior to death, we found downregulation of Hspa1a and Hspa1b with high downregulation of the regulatory fold change (Hspa1a 9.64 down, Hspa1b3.20 down). Considering its biological function, the unusual downregulation of HSP may represent suppression of the stress response [42] and misfolding of protein processing. These changes could further influence apoptosis [43, 44] and potentially change the composition and distribution of neurons in the brainstem, thus participating in SUDEP [8]. The mechanism of such downregulation remained unclear, although the coexpression analysis indicated that there is a lncRNA ENSMUST00000172531 adjacent to both *HSPA1A* and *HSPA1B* with a fold change of 2.61. It is likely that this lncRNA acts as an epigenetic regulating factor for the downregulation of these two genes. The KEGG analysis suggested several deregulated pathways, although they do not match the known mechanisms of S-IRA and SUDEP. Further study is still needed to confirm these findings.

Due to the difficulties in acquiring samples, studies on the expression profile and role of ncRNAs in SUDEPs are limited. In 2021, Leitner et al. reported an RNA sequencing study in SUDEP high-risk cases [45]. This study used hippocampal samples from mesial temporal lobe epilepsy surgery and found 55 dysregulated RNAs, including 37 mRNAs, 15 lncRNAs and 3 unknown RNAs. However, the published data only provided 20 mRNAs, and none of them overlapped with our dysregulated mRNAs or downstream targets of lncRNAs. Although the study used mTLE samples with a high risk of SUDEP, rather than real SUDEP, and the choice of hippocampal tissue is controversial, their study is the first and only attempt to systematically explore SUDEP from ncRNA prospects in humans. Christiansen et al. performed another epigenetic analysis by testing the methylation on the genome between sudden unexplained death cases and SUDEP. Their study found 6 different methylation regions near glutathione S-transferase (GST) superfamily genes, and the expression of *GSTT1* was negatively related to the methylation of GST [46]. In our study, we identified that *GSTT4* was downregulated (fold change 1.51, *P* = 0.027). By a similar mechanism to *GSTT1*, it may potentially influence the methylation of nearby genes and thus participate in SUDEP.

Our study has some limitations. First, we manually controlled the interval between the last seizure and sacrifice as 24 h, leaving long-term changes unavailable. Second, the tissue lacked anatomical precision, as the whole brainstem was used in the extraction of RNAs. Third, the heterogeneity of lncRNAs between humans and mice makes some of the results inapplicable for human studies.

## Conclusions

Abnormal expression of lncRNAs and dysregulated mRNAs was found in the DBA/1 SUDEP model compared to the normal control. Several RNAs, including Adora3, Lmnb2, Hspa1a, Hspa1b, Itrp1, Gstt4 and their related lncRNAs, were proposed as candidates related to SUDEP. The detailed mechanism of these dysregulated mRNAs and lncRNAs in SUDEP requires further study.

## Supplementary Information

**Additional file 1: Table 1. **Differentially expressed antisense lncRNAs and nearby coding gene. *The SUDEP group compared with normal group. **Table 2. **Differentially expressed lincRNAs and adjacent mRNAs. lincRNAs, long intergenic noncoding RNAs.*The SUDEP group compared with normal group.

**Additional file 2. ** Details of the corresponding probe and region of each lincRNA

## Data Availability

The original datasets analyzed in the current study are available in the Gene Expression Omnibus (GEO) repository (GSE160576). (https://www.ncbi.nlm.nih.gov/geo/query/acc.cgi?acc=GSE160576). The reference datasets include (X refers to any number or letter.): NCBI RefSeq database (NR_XXXXXX or NM_XXXXXX) (https://www.ncbi.nlm.nih.gov/refseq/) Ensembl (ENSMUSTXXXXXXXXXXX) (http://www.ensembl.org/index.html); mRNA or EST records in Genbank without coding evidence (AKXXXXXX) (https://www.ncbi.nlm.nih.gov/genbank/); UCSC known genes (ucXXXXXX.X) (http://genome.ucsc.edu/cgi-bin/hgTables/); "ultra-conserved region" among human, mouse and rat (uc.XXX) (http://users.soe.ucsc.edu/~jill/ultra.html); lincRNAs identified by John Rinn's group (Guttman et al. [47], Khalil et al. [48]) are coded as ‘humanlincRNAXXXX’ or ‘mouselincRNAXXXX’ in this research according to species. The detail of the corresponding probe sequence and region for each lincRNA is in supplementary material (see Additional file 2).

## Abbreviations

LncRNAs: Long noncoding RNAs
SUDEP: Sudden unexpected death in epilepsy
mRNA: Messenger RNA
GO: Gene ontology
PND: Postnatal day
AGSz: Generalized audiogenic seizures
S-IRA: Seizure-induced respiratory arrest
qRT-PCR: Quantitative real-time PCR
lincRNAs: Long intergenic noncoding RNAs
KEGG: Kyoto Encyclopedia of Genes and Genomes
ncRNA: None-coding RNA
HSPs: Heat shock proteins
