# Supplementary material for: Dysregulated long noncoding RNAs in the brainstem of the DBA/1 mouse model of SUDEP
Source: BMC Genomics. 2021 Aug 17;22:621. doi: 10.1186/s12864-021-07921-7 (PMC8369804; doi:10.1186/s12864-021-07921-7)
Supplement: Supplementary file 1 — Additional file 1: Table 1. Differentially expressed antisense lncRNAs and nearby coding gene. *The SUDEP group compared with normal group. Table 2. Differentially expressed lincRNAs and adjacent mRNAs. lincRNAs, long intergenic noncoding RNAs.*The SUDEP group compared with normal group. [file 12864_2021_7921_MOESM1_ESM.docx]

**Table 1 Differentially expressed antisense lncRNAs and nearby coding gene**

| **Seqname of lncRNA** | **Gene symbol** | **Fold change* (lncRNAs)** | **Regulation of lncRNA** | **Genome relationship** | **Nearby gene seqname** | **Nearby gene**  **symbol** | **Fold change***  **(mRNAs)** | **Regulation**  **of mRNA** |
| --- | --- | --- | --- | --- | --- | --- | --- | --- |
| AK032393 | AK032393 | 2.3386725 | down | natural antisense | NM_026670 | Zmym1 | 1.8163994 | down |
| AK012034 | AK012034 | 1.857779 | down | natural antisense | NM_010722 | Lmnb2 | 1.7845193 | down |
| uc007ovn.2 | Ifi27l2a | 1.7302904 | down | natural antisense | NM_194069 | Ifi27 | 1.8038755 | up |
| uc007ovn.2 | Ifi27l2a | 1.7302904 | down | natural antisense | NM_026790 | Ifi27 | 1.6717222 | up |
| uc007ovn.2 | Ifi27l2a | 1.7302904 | down | natural antisense | NM_194066 | Ifi27 | 1.6187848 | up |

Notes: *The SUDEP group compared with normal group.

**Table 2 Differentially expressed lincRNAs and adjacent mRNAs**

| **Seqname of lncRNA** | **Gene symbol** | **Fold change* (lncRNAs)** | **Regulation of lncRNA** | **Genome relationship** | **Nearby gene seqname** | **Nearby gene**  **symbol** | **Fold change***  **(mRNAs)** | **Regulation**  **of mRNA** |
| --- | --- | --- | --- | --- | --- | --- | --- | --- |
| AK013439 | AK013439 | 2.746032 | up | downstream | \| NM_001099328 \| \| --- \| | Zfp831 | \| \| 2.722746 \| \| --- \| \| \| --- \| --- \| | down |
| AK013439 | AK013439 | 2.746032 | up | upstream | NM_201617 | Gnas | 1.5863347 | down |
| ENSMUST00000172531 | 1110038B12Rik | 2.6065826 | down | downstream | NM_001142706 | Cfb | 7.2709559 | up |
| ENSMUST00000172531 | 1110038B12Rik | 2.6065826 | down | upstream | NM_010478 | Hspa1b | 3.1997402 | down |
| ENSMUST00000172531 | 1110038B12Rik | 2.6065826 | down | upstream | NM_010479 | Hspa1a | 9.6381486 | down |
| ENSMUST00000172531 | 1110038B12Rik | 2.6065826 | down | upstream | NM_013600 | Msh5 | 1.5513008 | down |
| NR_045838 | A730036I17Rik | 1.9323537 | down | upstream | NM_178404 | Zc3h6 | 1.5088414 | up |
| NR_004446 | H2-K2 | 1.8821076 | up | upstream | NM_011306 | Rxrb | 1.8016785 | down |
| ENSMUST00000172583 | H2-Q3 | 1.862996 | up | downstream | NM_001198561 | H2-Q7 | 1.5920826 | up |
| ENSMUST00000172583 | H2-Q3 | 1.862996 | up | downstream | NM_010391 | H2-Q10 | 4.0702323 | up |
| ENSMUST00000172583 | H2-Q3 | 1.862996 | up | downstream | NM_207648 | H2-Q6 | 1.9010357 | up |
| ENSMUST00000172583 | H2-Q3 | 1.862996 | up | upstream | NM_001267808 | H2-L | 2.1071947 | up |
| ENSMUST00000172583 | H2-Q3 | 1.862996 | up | upstream | NM_010380 | H2-D1 | 2.2766521 | up |
| ENSMUST00000172583 | H2-Q3 | 1.862996 | up | upstream | NM_010390 | H2-Q1 | 2.0065109 | up |
| mouselincRNA0188+ | mouselincRNA0188 | 1.8537734 | down | upstream | NM_031196 | Slc19a1 | 1.9678137 | down |
| ENSMUST00000120581 | Gm12307 | 1.8447556 | down | upstream | NM_030064 | Phf23 | 1.5970653 | down |
| ENSMUST00000173902 | Gm20495 | 1.7420145 | up | downstream | NM_001199967 | Gm11127 | 1.7084316 | up |
| ENSMUST00000173902 | Gm20495 | 1.7420145 | up | downstream | NM_010395 | H2-T10 | 1.7949279 | up |
| ENSMUST00000173902 | Gm20495 | 1.7420145 | up | downstream | NM_010398 | H2-T23 | 1.5619183 | up |
| AK041517 | AK041517 | 1.7385232 | down | upstream | NM_030221 | Nadsyn1 | 1.5768107 | down |
| ENSMUST00000126080 | 9830144P21Rik | 1.6401628 | down | downstream | NM_178404 | Zc3h6 | 1.5088414 | up |
| AK141112 | AK141112 | 1.5816486 | up | downstream | NM_001159671 | Rsph6a | 3.7486615 | down |
| ENSMUST00000181834 | Gm26744 | 1.5710093 | down | downstream | NM_023737 | Ehhadh | 1.5599215 | up |
| NR_040757 | 0610040F04Rik | 1.5610318 | down | downstream | NM_010585 | Itpr1 | 1.5445114 | up |
| ENSMUST00000169916 | Gm6728 | 1.548018 | up | upstream | NM_025806 | Plbd1 | 1.5226863 | up |
| ENSMUST00000149942 | Gm831 | 1.5451411 | down | downstream | NM_011214 | Ptpru | 1.6735317 | up |

Notes: lincRNAs, long intergenic noncoding RNAs.*The SUDEP group compared with normal group.
